# Subterranean Sympatry: An Investigation into Diet Using Stable Isotope Analysis

**DOI:** 10.1371/journal.pone.0048572

**Published:** 2012-11-05

**Authors:** Gillian N. Robb, Stephan Woodborne, Nigel C. Bennett

**Affiliations:** 1 Department of Zoology and Entomology, Mammal Research Institute, University of Pretoria, Pretoria, South Africa; 2 Natural Resources and the Environment, Council for Scientific and Industrial Research, Pretoria, South Africa; University of Utah, United States of America

## Abstract

In the Western Cape three species of mole-rat occur in sympatry, however, little is known about differences in their dietary preferences. Dietary composition of the three species; the common mole-rat (*Cryptomys hottentotus hottentotus*), the Cape mole-rat (*Georychus capensis*) and the Cape dune mole-rat (*Bathyergus suillus*) were examined using stable isotope analysis. Blood, fur and claw samples were collected from 70 mole-rats, in addition to several potential food items, to assess food selection of the three species under natural conditions. Overall there was a significant difference in the isotopic composition (δ^13^C and δ^15^N) between all three species and significant differences in their diet composition. There were also significant differences between tissues in all three species suggesting temporal variation in diet. The small size and colonial lifestyle of *C. h. hottentotus* allows it to feed almost 100% on bulbs, while the solitary and larger species *G. capensis* and *B. suillus* fed to a greater extent on other resources such as grasses and clover. *B. suillus,* the largest of the species, had the most generalized diet. However, overall all species relied most heavily upon geophytes and consumed the same species suggesting competition for resources could exist. We also showed a high level of individual variation in diet choices. This was most pronounced in *B. suillus* and *G. capensis* and less so in *C. h. hottentotus*. We demonstrate that stable isotope analysis can successfully be applied to examine dietary patterns in subterranean mammals and provide insights into foraging patterns and dietary variation at both the inter and intra population level.

## Introduction

In the Western Cape of South Africa three species of mole rat, the common mole rat (*Cryptomys hottentotus hottentotus*), the Cape mole rat (*Georychus capensis*) and the Cape dune mole rat (*Bathyergus suillus*) occur in sympatry. Mole-rats are subterranean and therefore harvest the majority of their resources from below ground. All three species are thought to be herbivorous, feeding on underground food resources such as geophytes which they gather by excavating their burrow systems, although little detailed knowledge is known about their dietary habits. Geophytes have been shown to be high in nutritional value and are also available as a resource for most of the year [1]. Many are also toxic and unpalatable to most other animals thus minimising competition with other small mammals [2]. *B. suillus* and *G. capensis* have also been reported to supplement their diet with aerial vegetation such as grasses, by pulling the plants down into the burrow [3].

When similar species coexist in the same habitat it is predicted that resource partitioning occurs. There are three main ways in which species may achieve this; spatial separation [4], temporal avoidance [5] and through dietary differences [6]. In effect species are able to make use of the resources in their habitat differently, for example, through alternate foraging strategies so that they are able to occupy different niches within the same environment [7].

Although similar, the three species exhibit a number of morphological and behavioural differences. First, the three species differ in their social systems. *B. suillus* and *G. capensis* are solitary, whereas *C. h. hottentotus* is social, occurring in colonies of between 2 and 14 individuals [8], [9]. Second, the three species differ in body mass, the Cape dune mole-rat, *B. suillus* is by far the largest weighing 504 to 1291 grams in this study, *G. capensis* ranged from 34 to 287 g, while *C. h. hottentotus* is the smallest of the species with a body mass range of between 27 and 84 g. These differences are likely to influence dietary requirements, due to the varying energetic demands imposed by different sizes and also the type of foraging strategies that are employed by the mole-rats [10].

Optimal foraging theory predicts that animals will choose resources based on maximising their net energy gain in the shortest possible time [11]. Resource use therefore depends on factors such as abundance and encounter rates as well as nutritional value [12]. Mole-rat foraging behaviour can be confined by the limits of the burrow system, which may increase the importance of encounter rates as individuals are more restricted in the potential resources they have access to. There is also an increasing energetic cost associated with foraging to new areas. Moving underground has been reported to be 360–3400 times more costly than travelling over the same distance above ground [13]. It has been suggested that increased foraging expenditure, coupled with a non-directed search pattern, has led subterranean mammals to be dietary generalists and that individuals should collect all consumable foods they encounter [14]. However, some subterranean mammals have been shown, despite their overall broad diet, to show selectivity and consume some foods more than others [15]. For example in a captive feeding study on the silvery mole-rat (*Heliophobius argenteocinereus*) a preference was shown for food which had a high sugar content [16].

Traditional methods of examining diets in mole rats have used techniques such as analysing stomach contents, identifying food items in food stores and captive feeding trials [17], [18]. However, these methods have some drawbacks, for example stomach content analysis only shows diet over a single sampling event, representing a relatively short time period, and can also be biased towards certain food types. For example Norris (1943) found that in sheep different forbs were retained in the stomach for different lengths of time according to their digestibility, succulent plants passed through the stomach quickly while dry woody plants were slower [19]. Examination of food stores is restricted to items stored and does not take into account items eaten *in situ* when found, while captive feeding studies may not necessarily reflect natural behaviour. In this study we use an alternative non-invasive method, stable isotope analysis, which offers a potential solution to a number of these problems [20]. This technique could be particularly beneficial for underground species such as mole rats where direct observation of feeding is impractical. Measuring the stable isotope ratios of a consumer’s tissues can accurately reflect all food sources in the diet since the isotope ratio is derived from all nutrients assimilated and depending on the turnover of the tissue chosen for analyses, reflects diet over variable temporal scales [20]. Different food items can often have distinct stable isotopic signatures, and these are incorporated into the tissues of consumers in a predictable manner [21]. Thus, by measuring the stable isotope ratios of consumer tissues, it is possible to assess the relative importance of isotopically distinct food sources. We can also use isotopes to calculate the isotopic niche. Isotopic values for animals can be plotted in bivariate space called d space, where different axis represent different isotopes [22]. The isotopic niche is then the position occupied by the tissue within this space.

We used carbon and nitrogen isotopes to assess the isotopic composition of the animal tissues as well as potential food items. Carbon can be particularly useful for discriminating between different plant sources in the Western Cape as it allows for distinction between plants that use the C_3_ photosynthetic pathway, such as bulbs and tubers and those which use CAM or a C_4_ photosynthetic pathway, such as C_4_ grasses [23]. Different tissues have been shown to turnover (incorporate isotopic signatures) at different rates, and thus the utilisation of different tissues provides an indication of diet over different temporal periods [24]. Three different tissue types were chosen for the study, whole blood, fur and claws.

One problem associated with predicting diet composition from a number of sources using mixing models is the issue of metabolic routing. Metabolic routing occurs when different macronutrients are metabolized by different routes as they are incorporated into the tissues which can lead to overestimation or underestimation of certain food items [25]. Knowledge of the macronutrient composition of the diet is useful but not always obtainable. However, this problem can be alleviated to some extent by running concentration-dependent models as in this study. We also included tissue specific discrimination rates which should help reduce any bias.

In this study we aim to investigate the diets of three species of mole-rat in their natural environment, using stable isotopes to examine the importance of different food sources. We will assess between species differences as well as the amount of individual variation that exists within the species themselves. We will also test for any differences between tissues to identify any changes in diet during different periods of the year.

## Materials and Methods

### Ethics Statement

All work carried out in this study was approved by the University of Pretoria Animal Use and Care Committee (permit number EC060-09) and with relevant permits from the Western Cape Nature Conservation Board (Permits to collect fauna and flora specimens for scientific research: permit numbers AAA-004-000403-0035 (fauna) and AAA004-00028-0028 (flora) and with permission from the landowner (J. Duckitt).

### Data Collection

The study was conducted at ‘Waylands’ farm, 6 km south-east of Darling in the Western Cape, South Africa (33.25°S, 18.25°E). The farm has a diverse Fynbos flora, including a variety of geophytes. During the summer of 2010 a total of 70 mole rats (9 *Batherygus suillus*, 17 *Cryptomys hottentotus hottentotus* and 44 *Georychus capensis*) were trapped using modified Hickman live traps baited with sweet potato [26].Once captured, tissues samples were collected from all animals. Prior and post sampling animals were held for a short duration in suitable containers with appropriate food and bedding provided. A small sample of blood was removed from the hind foot, 1–2 claw(s) from the hind feet and a small patch of fur from the back, just above the tail. Results for comparison between tissue and species used the average claw result for each individual. Whole blood is likely to represent diet over a number of weeks (half-life of tissue carbon and nitrogen was 24.8 and 27.7 days, respectively, for rats) [27], fur a longer time period (half-life of tissue carbon and nitrogen was 65.1–70.2 and 79.9–85.4 days, respectively, in rats) [28] and claws a number of months. Based on the growth rate of claws in rats of 0.04 mm per day [29], the claw tip is likely to represent a period of approximately 50 days, about 3–4 months prior to the time of sampling. The stable isotope composition of fur and claws represent the period during which the fur and claw is grown and then since the tissues are inert, remains unchanged [30]. As little is known about the moult cycle of mole-rats we cannot predict precisely which time period the fur will represent in this study. Animals were also sexed and biometric measurements (body length, head length etc.) and body mass recorded. Following sampling, animals were released back into their burrows. In addition samples were also collected from euthanized animals collected from the same farm as part of other ongoing research projects during the summer of 2011.

Potential food items were collected in close proximity to the burrows from all areas where animals were trapped during both the summer and winter of 2010. Collecting food items in two different periods of the year allowed us to detect any changes in the signatures of the plants throughout different times of the year. A total of 14 different species of bulb and corms were collected with the number of species between fields varying from between 3–10. Previous diet studies have shown mole-rats consume mainly geophytes [17, 31 32], collection was therefore restricted to bulbs, corms and tubers; in addition grasses and clover were also collected. Clover was collected as this was observed to be present in food stores during the sampling period. Items from food stores were also gathered from burrow systems that were being excavated as part of other projects. Where possible, plants were indentified to species level. Samples were then dried and stored in paper bags.

### Stable Isotope Analysis

All samples (blood, fur, claws and plants) were dried in an oven at 65+ °C for 48+ hours to a constant mass. Parts of the plants known to be consumed were used in the isotope analysis, for examples husks which are removed by the mole rats were removed and the inner bulb only sampled. Samples were then weighed into tin cups and run in a continuous flow isotope ratio mass spectrometer (DeltaV IRMS coupled with a Flash 1200 elemental analyser with a ConFloIV interface, all instruments from ThermoFisher, Bremen, Germany) for stable isotope analysis. For *G. capensis* and *C. h. hottentotus* two claws were used and run as separate samples. For *B. suillus*, as claws were larger, a single claw was cut into between 4–10 sections depending on the size. This allowed time series data to be collected from a single tissue. Both carbon and nitrogen isotope ratios were measured and samples were run in duplicate to check for reproducibility. Standards were also included in every run to correct raw values. Results for stable isotope ratios are expressed using the standard δ-notation as ‰, according to the following:
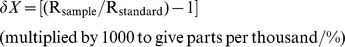
where X is ^13^C or ^15^N and R is the corresponding isotope ratio ^13^C/^12^C or ^15^N/^14^N and R_standard_ is the international standard for carbon (PDB) and for nitrogen (AIR) and thus defined as 0‰.

### Isotope Modelling

Diet compositions were calculated for the three species using SIAR (Stable Isotope Analysis in R), to examine reliance on different food groups. SIAR produces a range of solutions regarding the proportional contribution of each food source to the diet for every individual. It utilises a Bayesian approach and has the advantage that it is able to incorporate many sources of variability and multiple dietary sources. True probability-density functions are produced, hence, the median value of the probability–density function represents the most probable solution and this number is used in subsequent analysis and presented in the results. Food items collected from the same field where the animal was trapped were used to predict diet choice. The type of food items did differ between different fields so analysis was restricted to those the animal would have been potentially able to access within the field where they were present. Bulbs and corms, identified to species level and abundant in the location, were entered into the analysis as individual food items, in addition to clover, grass, tubers and a small number of unidentified bulbs. Average signatures with standard deviations were used for the last four groups.

**Figure 1:**
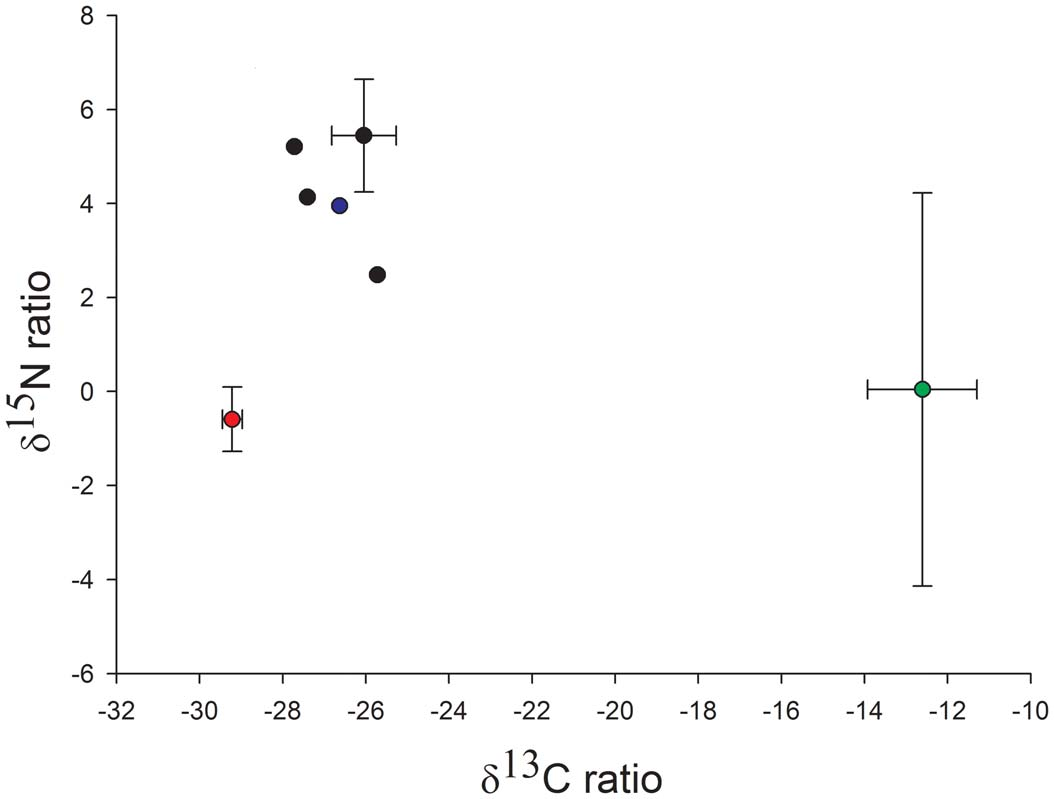
Example of isotopic values for food items found in one field where mole-rats were sampled. Green indicates average grass signature (± SD), red average clover signature (± SD), blue average tuber (only one species in this field) and black dots bulb and corm species (± SD).

Food sources were checked for isotopic separation between the different groups prior to analysis. Grass could be distinguished from all groups based on the carbon signature. Although there are also C_3_ grasses present in the Western Cape, we show that the grasses we measured in the diet of the mole-rats are a C_4_ source, represented by a more 13C-rich composition (between −11 to −14), making the distinction with bulbs and tubers possible. Clover could be distinguished from bulbs, corms and tubers by a less 13C-rich composition combined with an overall less 15N-rich composition. Bulbs, corms and tubers were most similar in their signatures. However, there was no overlap between the identified bulbs and corms, unidentified bulbs and tubers entered into the models (Example of food sources used in one trapping area for the SIAR models are shown in Figure 1). To examine diet at a species rather than an individual level the mean value for the proportions for each food source were calculated from the diet composition results for the individuals.

Tissue specific discrimination rates were added to the models (based on captive studies, G. Robb *et al*. unpublished data). Species specific rates were applied for *C. h. hottentotus* for all tissues and for claws and fur for *G. capensis*. However, for *G. capensis* blood and all tissues for *B. suillus* an average ‘mole-rat’ discrimination rate was used (Table 1) Concentration dependent models were applied in order to incorporate variation in elemental concentration dependence in the different sources. This is calculated as the mean % C and mean %N (+/− standard deviation) for each of the food sources in the model.

**Table 1:** Discrimination values used to correct stable isotope data.

Species	Blood		Claw		Fur	
	C	N	C	N	C	N
*C. h. hottentotus*	1.3	5.0	2.3	5.3	2.6	5.5
*G. capensis*	1.8	4.9	2.2	5.1	2.2	6.4
*B. suillus*	1.8	4.9	2.4	5.1	2.3	6.1

## Results

### Differences between Species

**Figure 2:**
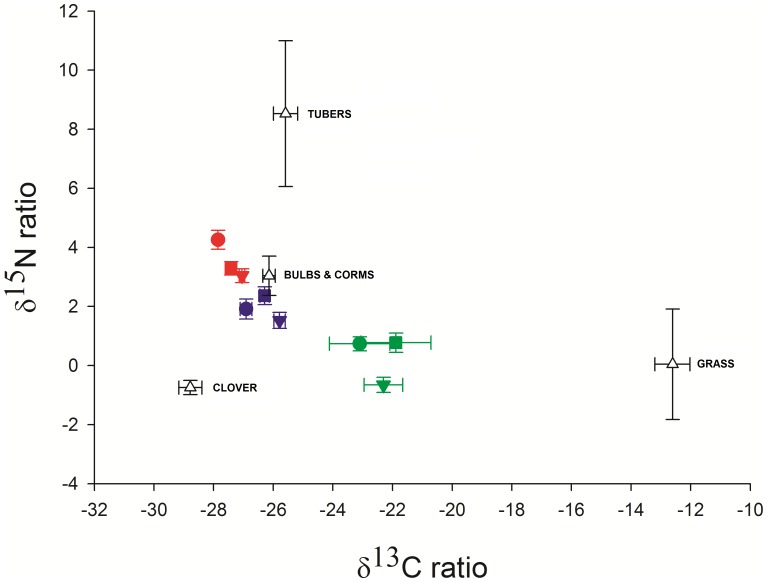
Mean stable isotope compositions of the three species (*C. h. hottentotus = *red, *G. capensis = *blue and *B. suillus = *green). Tissues are shown by shape of symbols (circle = blood, square = claw and triangle = fur). Error bars represent ±1 standard error. Mean values (+/− se) are also shown for the main food sources (white triangles). All animal tissues have been corrected using appropriate discrimination values.

Overall differences between the three species in their carbon and nitrogen isotope compositions were examined to assess the degree of overlap in their dietary niches. As tissues have specific discrimination rates all samples were adjusted for appropriate tissue and species discrimination rates prior to the analysis. Multivariate general linear models showed significant differences between species for all tissue types (Table 2). Due to the small number of samples collected for blood in *C. h. hottentotus* (only 3), this species was only included when examining species differences using claw and fur samples. *B. suillus* showed the most distinct isotope values (Figure 2), with a less 15N-rich composition and a more 13C-rich composition than *G. capensis* or *C. h. hottentotus*.

**Table 2:** Multivariate general linear models for differences in carbon and nitrogen stable isotope composition between the three species.

Tissue	F	df	P
Blood	30.050	2, 30	<0.001
Claw	26.586	4,114	<0.001
Fur	28.103	4,110	<0.001

Layman’s metrics were calculated for all species using claws and fur [33]. NND (nearest neighbour distance) can be used to assess the overall similarity of trophic niches among individuals within a population. *B. suillus* had a greater NND for both tissue types than either *G. capensis* or *C. h. hottentotus* (Table 3), indicating a greater difference among individuals within this species. *C. h. hottentotus* showed the smallest NND showing the greatest similarity between individuals. *C h. hottentotus* is social and therefore a number of individuals were from the same colony, sharing the same burrow system and with access to the same resources, consequently a more similar signature between individuals is probable.

**Table 3:** Layman metrics and standard ellipses for claw and fur samples.

Tissue	δ N range	δ C range	Mean NND	Convex hull	Area of ellipse	Sample size
**Claw**						
*B. suillus*	3.52	12.15	1.62	11.90	6.52	9
*G. capensis*	6.73	3.88	0.42	18.70	6.34	38
*C. h hottentotus*	2.42	1.42	0.29	2.39	0.99	16
**Fur**						
*B. suillus*	2.54	5.61	0.71	4.22	2.52	9
*G. capensis*	6.62	4.44	0.45	19.99	5.25	39
*C. h. hottentotus*	2.76	1.30	0.21	2.18	0.88	16

Ellipses were calculated to provide a measure of a population’s total niche width (Table 3). These were produced using the program SIAR by fitting a standard ellipse to the bivariate (carbon and nitrogen) data using maximum likelihood estimators. Using claw samples, *B. suillus* and *G. capensis* were similar in size, however, with fur samples *G. capensis* showed a much larger niche width. The larger size of the *G. capensis* fur ellipse could partly be due to increased sample size, however ellipses can be corrected for small sample size and are also reported to be less subject to the problems of sample size than other methods such as convex hulls [34]. Both the ellipses and the mean and standard errors (Figure 2) show the narrower isotopic niche width of fur and claw samples in *C. h. hottentotus* compared with the other two species. The size of the convex hulls, encompassing all the data points, is also provided in the table as a comparison to the ellipse data.

**Figure 3:**
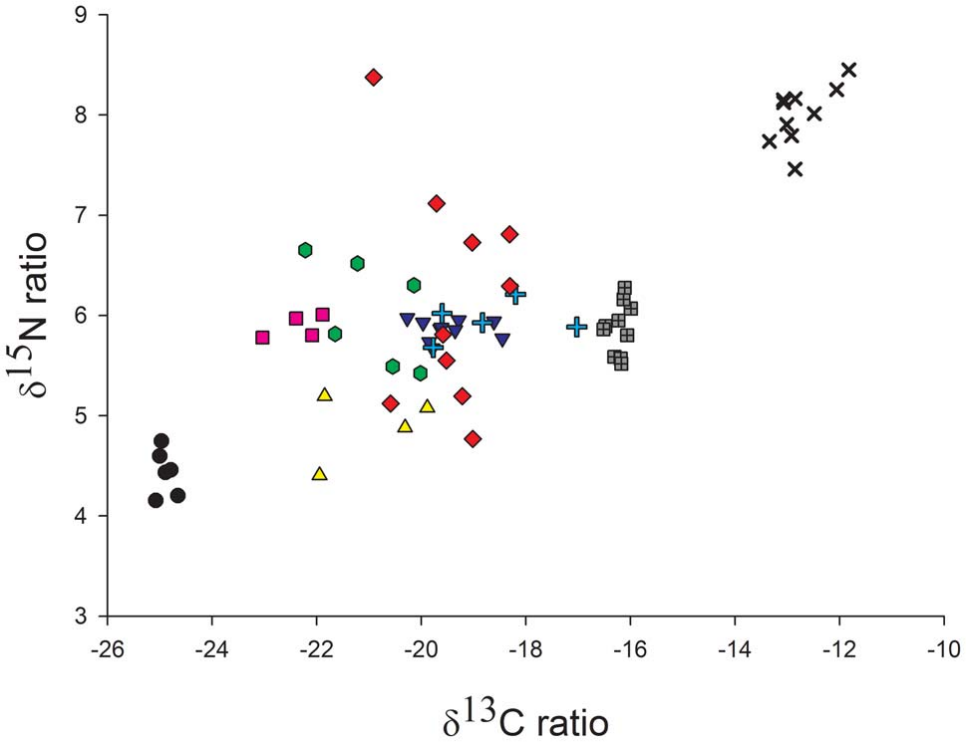
Carbon and nitrogen isotopic values for claw sections from the Cape dune mole-rat, *B. suillus*. Each colour and symbol represents a single individual showing inter and intra variation in individual diets.

*B. suillus* claws were large enough to allow sectioning for analysis of a time series. Each section was between 1–2 mm in length and therefore, if rat claw growth rates are applied, this equates to between 25–50 days growing time. These were examined to assess whether variation in isotope compositions observed in the different sections of claws could be better explained by differences between individuals or within individuals. The results show that nearly all variation could be explained by differences between individuals and that within individual variation contributed little to the overall variance (Figure 3, Table 4). This suggests that although there may be differences between individuals in their diet choice, the same individual continues to eat a similar diet over the time period represented in the claws.

**Table 4:** ANOVAs show significant differences between the means of the isotopic values of claws in individuals of *B. suillus*, with most of the variance explained by between individual rather than within individual differences.

		Sum of Squares	Variance explained %	F	*P*
C	Between Groups	762.452	0.995	213.327	<0.001
	Within Groups	25.019	0.005		
N	Between Groups	60.318	0.966	28.760	<0.001
	Within Groups	14.681	0.034		

### Differences between Tissues

Differences in the three tissues sampled within the same species were examined. Individual tissues, with different turnover rates, can be used to examine temporal changes in diet. Tissue specific discrimination rates were applied for blood, fur and claw, to remove any differences due to the physiology rather than diet in the signature. Multivariate repeated ANOVA showed significant differences between the tissues in all three species (Table 5). A repeated design was used as a single individual was sampled for all three tissues. Blood, fur and claw were analysed for *B. suillus* and *G. capensis*, while due to the small number of samples collected for blood, only fur and claw were analysed for *C. h. hottentotus*. Univariate repeated ANOVAs on the single isotopes, carbon and nitrogen, were carried out with individual as the repeated measure. Results showed there were significant differences for all species in carbon and significant differences in *B. suillus* and *G. capensis* for nitrogen but not in *C. h. hottentotus* (Table 5).

**Table 5:** Multivariate and univariate general linear models for differences in carbon and nitrogen stable isotope composition between the three tissues.

Multivariate repeated anova	Wilks’ λ	F	Df	*p*
*Batherygus suillus*	0.008	78.330	4,32	>0.001
*Georychus capensis*	0.217	25.799	4,90	>0.001
*Cryptomys h. hottentotus*	0.330	12.188	2,13	0.01
**Univariate repeated anova δ^15^C**	**F**	**df**	***P***	
*Batherygus suillus*	948.37	2, 7	>0.001	
*Georychus capensis*	28.240	2,23	>0.001	
*Cryptomys h. hottentotus*	24.649	1,13	>0.001	
**Univariate repeated anova δ^15^N**	**F**	**df**	***P***	
*Batherygus suillus*	455.660	2,7	>0.001	
*Georychus capensis*	9.200	2,23	>0.001	
*Cryptomys h. hottentotus*	0.627	1,13	0.443	

### Importance of Size and Sex in Determining Isotope Composition

Three measures; body mass, body length and sex were used to examine if there were any differences in diet based on gender or size, which might also indicate social or reproductive status in colonies of social *C. h. hottentotus*. Multivariate GLMs were run for the three species for each tissue type separately. Carbon and nitrogen were included as the dependent variables, sex as a fixed effect and body mass and body size were incorporated as covariates. Post-hoc tests were done following all models to examine the importance of the individual isotopes. Blood and claw samples showed no significant relationships with any of the variables, while two significant results were found with fur. In *B. suillus,* body mass had a significant effect on carbon isotope compositions (F_1,9_ = 6.51, *P = *0.038), with heavier individuals exhibiting more 13C-rich composition. While in *C. h. hottentotus* body mass had a significant effect on the nitrogen composition, with heavier individuals showing a more 15N-rich composition (F_1,16_ = 6.718, *P = *0.021).

### Differences between Years

We then assessed if there was any difference between animals trapped in the same fields over different years. *G. capensis* were collected from the same field in the summer of both 2010 and 2011. However, there was no significant difference in the carbon and nitrogen isotope composition in the fur (MANOVA, F_2,42_ = 1.082, *P = *0.348) or the claws (MANOVA, F_2,40_ = 0.126, *P* = 0.882).

### Dietary Composition

Diet compositions were calculated for the three species using SIAR (Stable Isotope Analysis in R), to examine reliance on different food groups. Tissues used in the analysis were from the summer of 2010, blood therefore likely represents diet in the summer, claws from the spring/early summer (based on turnover rates in other rodents) and fur a longer term diet choice. Although as the moult cycle remains unknown we cannot say which exact temporal period the fur will represent. Food sources were divided into 3 main groups; geophytes, clover and grass, all of which were abundant in all locations. Grass and clover were present in all fields where mole-rats were trapped and therefore available as a potential resource to all animals. Bulbs, corms and tubers were also present in all fields; however the number and type was variable according to the location.

**Figure 4:**
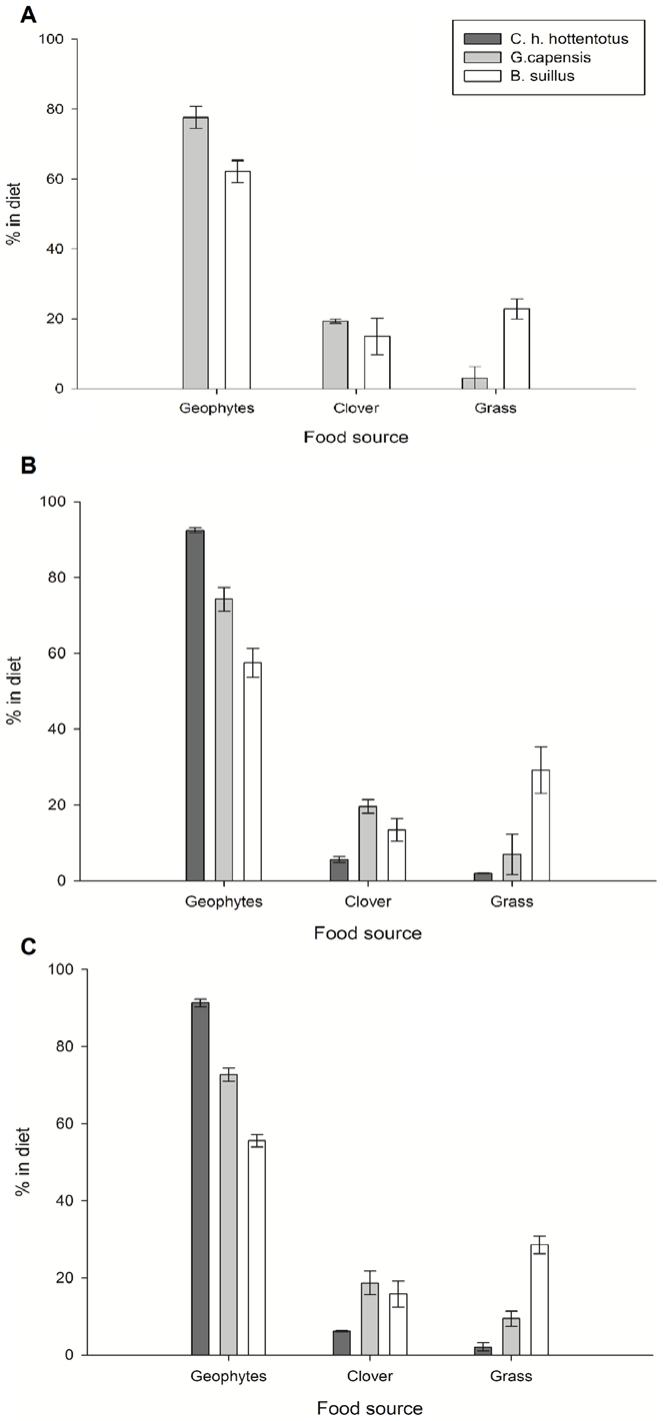
a–c. Relative consumption as predicted by SIAR of the three main food groups in each of the different tissues sampled (A) blood, (B) claws and (C) fur for *B. suillus, G. capensis* and *C. h. hottentotus.* **a.** Mean (+/− s.e) relative consumption of the three main food groups from blood samples of each respective species. **b.** Mean (+/− s.e) relative consumption of the three main food groups from claw samples of each respective species. **c.** Mean (+/− s.e) relative consumption of the three main food groups from fur samples of each respective species.

Figures 4a–4c show the relative reliance on the three main food groups identified. The percentages represent the mean (+/− SE) value for each species based on the individual outputs generated in SIAR for each of the food sources. Median tests (Table 6) show significant differences between all groups for all three species in fur and claws. While in blood, comparing diet composition between *G. capensis* and *B. suillus* only, significant differences were found between the use of geophytes and grass but not in the use of clover.

**Table 6:** Results of Median tests showing differences in dietary compositions between species (shown in Figure 4).

Tissue	Median test	Geophytes	Grass	Clover
Blood	Chi square	11.65	8.09	0.08
	P value	0.001	0.004	0.776
Claws	Chi square	24.374	20.817	9.511
	P value	<0.001	<0.001	0.009
Fur	Chi square	27.45	26.38	12.94
	P value	<0.001	<0.001	0.002

Blood represents samples from *G. capensis* and *B. suillus.* Claws and fur between all three species.

*B. suillus* has the greatest reliance on grass in all tissue types analysed and the least reliance on geophytes (less than 62% of the diet) compared with the other species. *G. capensis* showed a diet intermediate between *C. h. hottentotus* and *B. suillus* in their use of the three resources, in that although the largest component of their diet was geophytes, they also consumed relatively large amount of clover and to a lesser extent grass. The food store during a burrow excavation of *G. capensis* contained a number of small bulbs and corms but also a large amount of clover supporting the use of this as a food item. The diet of *C.h. hottentotus* was mainly composed of geopyhtes, with a small percentage of clover and very little reliance on grass. Overall all three species had geophytes as the largest component of their diet. Dietary composition was similar between tissues for all three species. However, there was a small difference between blood and the other two tissues in both *G. capensis* and *B. suillus*.

Within the identified bulbs and corms both *G. capensis* and *C. h. hottentotus* do not show a clear preference for a single species, although least preference by both species is shown for *Ornithogalum thysoides*, approximately 13–14% of the total bulb and corm consumption in both species and highest preference for *Romulea spp*. (approximately 25–41%). *B. suillus* showed the greatest preference for *Ammocharis longifolia* (42–60% of the bulb and corm diet depending on tissue type). This is a large bulb, 100–150 mm in diameter, and therefore might be favoured over smaller sized bulbs. This species of bulb also grows more frequently in sandy soils which are also favoured by *B. suillus*. *Zantedeschia aethiopica* which was eaten by all three species contributed most to the overall bulb and corm diet of all the mole-rat species together. Whereas all three food groups, geophytes, grass and clover were abundant in all fields the availability of a particular species did differ. During sampling we observed that *Romulea* was very abundant, with multiple species being observed in the same area. Interestingly, *Ornithogalum thysoides* when observed was less abundant and therefore probably encountered less often during foraging.

### Individual Variation in Diet Composition

Diet composition predicted from SIAR showed the variability that exists between different individuals, even within the same species in the same field. Proportions of the main food groups altered greatly. For example within *B. suillus,* grasses contributed between 2 and 64% to the diet as revealed by claws and 18–47% of the diet as revealed by fur. While in *C. h. hottentotus*, the species which consumed the least amount of grass, individual variation ranged from 1.5–2.7% in claws and 1.4–3.2% in fur. There was also variation in the importance of the other main sources, for example the percentage of clover in the diet estimated using fur samples, ranged from 7–28% in *B. suillus*, from 3–17% in *C. hottentotus* and from 3–34% in *G. capensis*. While diet compositions predicted from fur samples for bulbs and corms found an individual range of 39–48 for *B. suillus*, 81–95% for *C. h. hottentotus* and 24–99% for *G. capensis*.

## Discussion

We found there was a significant difference in the isotopic composition of the three species of mole-rat on both the d^13^C and d^15^N axes, and that these differences were maintained over different temporal scales. *B. suillus* showed the most distinct isotopic composition with a more 13C-rich composition and less 15N-rich composition than the other two species. Whilst the distinction between *C. h. hottentotus* and *G. capensis,* although significant, was less pronounced showing the closest overlap in dietary niche. Overall *C. h. hottentotus* exhibited the narrowest dietary range, with a smaller total niche width in both claw and fur samples. This was further supported by the dietary composition results which showed over 90% of their diet consisted of geophytes. *B. suillus* showed the greatest range in isotope variability, reflecting their greater reliance on sources such as grasses, clover and tubers in addition to bulbs and corms.

The significant differences between tissues for all species tell us that diet is not constant over time but changes, implying that they are isotopic generalists, i.e. the relative proportion of different food sources change over time. This is in contrast to dietary generalists who can show a constant, but wide, diet over time. We might predict that reliance on the different food sources would change throughout the year as their availability and nutritional value alters [35]. Underground storage organs like bulbs, corms and tubers are likely to have to the greatest nutritional value in April/May, when they have the greatest amount of energy stored to last through the winter, whereas by October/November they are likely to be of lower nutritional value as energy has been diverted into flowering. A previous study found *B. suillus* ate more bulbs in April compared with October [17]; we might therefore predict that a greater proportion of the diet would be predicted to come from bulbs, corms and tubers in blood, representing February/March, than in longer term tissues such as claws and fur and this is what was observed. In both *G. capensis* and *B. suillus* the greatest use of geophytes and least reliance on grass and clover was found in the diet composition predicted from blood compared with both fur and claws, indicating that geophytes may become more important in the diet during the late summer months compared to earlier in the year. We also found variation in how much the diet of an individual changes over time. For example the time series data of *B. suillus* claws shows that while the isotopic signature of some individuals remains relatively constant over time others show greater variation and are likely to switch between different food sources to a larger extent (Figure 3).

It is possible the differences between tissues are not due to variations in diet composition but due to changes in the base of the food web (i.e. differences in the bulbs, tubers etc. over different temporal periods). We sampled food items in both summer and winter and little difference was found in the isotopic signature of the food items collected from the same field in both time periods. For example the signature of *Zantedeschia aethiopica* changed from C −25.02, N 2.77 in summer to C −25.71, N 2.48 in winter, and clover in the same field changed from C −29.49, N −0.79 in summer to C −29.11, N −1.16 in winter.

Nutritional stress has been shown to cause nitrogen enrichment [36] but has little or no effect on d^13^C values. Differences could therefore be a result of the physiological condition of the animals during the time the tissues represent. As a change in carbon isotope composition was also observed physiological condition is also unlikely to explain the variation. This suggests the differences between tissues are more likely due to differences in dietary composition. Further analysis into diet using SIAR supports this, as the relative proportions of the different food groups alters between different tissues types. The main shift between the three tissue types in *G. capensis* was in the carbon isotope composition, with it becoming more depleted from fur, to claws, to blood. This correlates with a decreased reliance on grass (13C-rich carbon) between blood and the other two tissues. *B. suillus* also showed a decrease in the amount of grass in the diet in the blood samples, although this was less pronounced than in *G. capensis.* Tissue differences in *C. h. hottentotus* were also mainly due to a change in carbon isotope composition, following the same patterns as in *G. capensis.* However, we did not have enough samples for blood, only 3, to detect any changes in diet composition between blood and the other two tissues.

**Figure 5:**
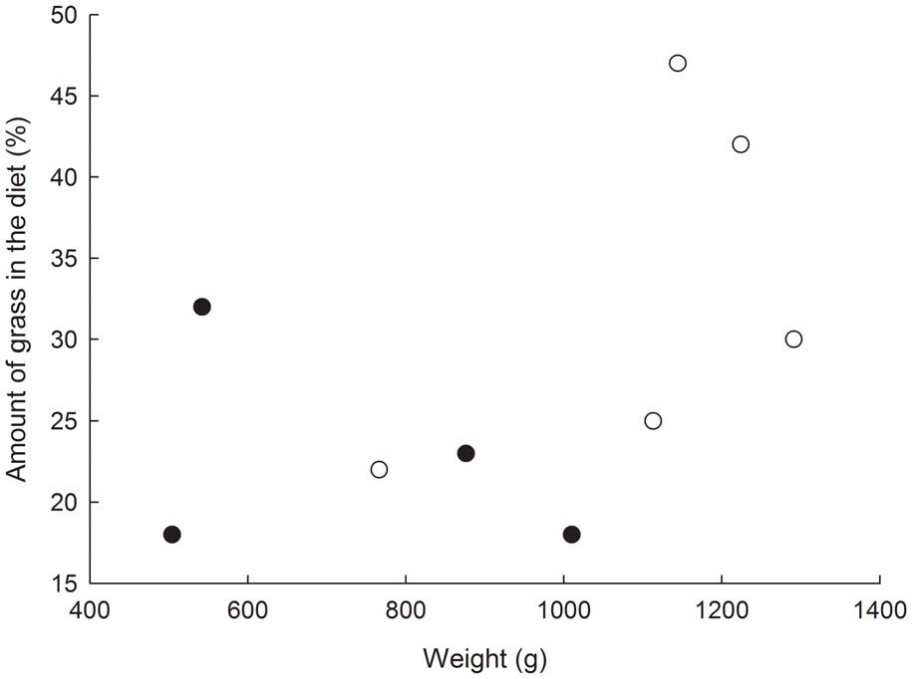
The percentage of grass in the diet as calculated by SIAR in relation to body mass and sex in *B. suillus*. Females are represented by filled black circles and males by open white circles.

We might predict that both sex and size could affect diet composition and therefore stable isotope compositions, however, overall we found little effect of either of these variables on carbon or nitrogen. One relationship observed with fur samples was that *B. suillus* with larger body masses had more 13C-rich carbon isotope compositions. This indicates that larger individuals relied more heavily upon grasses compared with smaller individuals. This hypothesis can be tested using the data for the diet composition predictions (Figure 5). There is a trend of larger individuals, which are also mainly male, to have a greater percentage of grass in their diets. However, a larger sample size would be required in order to further substantiate this conclusion. One possible reason for increased grass consumption by larger individuals could be that the increasing food requirements by larger individuals cannot be fully met within the confines of the burrow system through eating geophytes alone, but has to be supplemented with grasses to a greater extent, while in smaller individuals energetic costs could be met mainly from consumption of bulbs, corms and tubers which may be more nutritionally beneficial and a preferred food source [1].

Results of the relative use of different food groups estimated using SIAR can be compared with a previous study of diet in mole-rats using stomach content analysis on the same study site in the 1980s [17]. This study examined the same species and also found that *B. suillus* ate the greatest amount of green forbs and grass (20–85% of the diet). The range for individuals estimated using stable isotopes in our study for grass and clover combined was from 24–66%, with an average across all tissue of between 37–44%. Beviss-Challinor (1980) also estimated *G. capensis* to have between 4 and 15% green forbs and grass in their diet. We found a much larger range for this species, with an average for all tissue of 22–27% and would therefore suggest that these sources of food are of greater importance than previously predicted for this species. *C. h. hottentotus* were also analysed and found to feed 100% on bulbs [17]. Although we also found a high percentage of geophytes in the diet in this species in our study (92%), we also estimated a small use of other food sources as well. The differences between the findings from the Beviss-Challinor study and our results could be due to changes in the availability of different resources between the different years, the shorter time scale reflected in stomach analysis compared to stable isotope analysis or due to differences in sample size (18 animals were sampled in the Beviss-Challinor study compared to 70 in the present one).

In this study all three species were predicted to consume clover as part of their diet, this has not been previously identified as an important food source for mole-rats. However, the nitrogen content of clover compared to the other sources was higher and it is possible that mole-rats, being herbivorous, may choose to feed on clover as it provides a good source of nitrogen. We also assessed preferences for particular species of geophytes when examining diet compositions. However, differences in the abundance of species makes it is difficult to distinguish between a chosen preference by the mole-rats for a particular species and consumption rates being merely an outcome of availability and encounter rates.

The majority of studies assessing foraging have tended to focus on the population level, however, there is increasing evidence for the importance of individual variation that exists [37]. Bolnick (2003) highlights how understanding individual variation can have important implications for our understanding of ecological and evolutionary processes [38]. The results from the diet composition analysis enabled us to examine the level of variation that exists in the diets of individuals. Variation was found across all three species, but was especially pronounced in *B. suillus* and *G. capensis* and less so in *C. h. hottentotus,* as also shown by the size of the ellipses and convex hulls. We predict this could be partly a result of their social systems, varying from solitary to social living in colonies. Individuals living in colonies, like *C. h. hottentotus*, are likely to have access to similar resources, foraging in the same burrow system, and therefore have less variability between individual diets than between solitary animals living in individual burrow systems. However, even if we account for the fact that some of the *C. h. hottentotus* individuals were from the same colony, the differences between individuals overall were still smaller than in the other two species.

The results from the multiple claws analysed for *B. suillus* suggest that although there is a low within individual variation, there is variation between individuals. Some individuals appear to be more constant in their diet choices, while others consume a wider variety of items over time. Although this study site is particularly rich in geophytes and all burrows had access to all three food groups, there could be differences in the availability of resources between individual burrow systems.

Although all three species can occupy the same field and be found in close proximity to each other, within that space there can be a degree of spatial separation between *B. suillus* and the other two species. *B. suillus* was found more commonly in sandy less compact soils of the field, whereas *C. h. hottentotus* and *G. capensis* were found in the more compacted loam and clay soils. Soil type is likely to affect the composition of plants that grow and therefore spatial differences could also lead to variation in encounter rates of food items. This could partly explain the closer similarity in diet between *G. capensis* and *C. h. hottentotus* when compared with *B. suillus*. Distinguishing between the observed differences in diet being a by-product caused by spatial separation or a mechanism of coexistence could be difficult. For example Comparatore, Cid & Busch (1995) found that two species of sympatric tuco-tucos *(Ctenomys)* distribution was related to soil characteristics and that this may have led to the observed differences in dietary habits as individuals fed on food sources within their own home ranges [39]. Food availability of the different resources was not quantified in this study; however all food sources, bulbs, corms and tubers, grasses and clover were located in all fields and were observed to be abundant. Another study conducted on the same species on the same farm carried out vegetation transects across fields and found that there was no significant change in bulb numbers and masses along transects [17]. Reichman and Jarvis (1989) also conducted research on the same farm and found that against their prediction, mole-rats did not inhabit those areas of the site with greater than average plant biomass and that neither *C. h. hottenotus*, the bulb specialist, nor *B. suillus*, which consumes significant quantities of aboveground plant material, chose areas especially rich in their diet specialties [40]. They also suggested that *G. capensis* may be able to inhabit areas the other two species have already exploited and that both *G. capensis* and *C. h. hottentotus* may be able to forage for bulbs in areas already used by *B. suillus*. However, there was no direct evidence for this [40].

Overall, although food items utilized by the three species were similar there was still evidence of differences in their diet compositions. All three species relied upon geophytes as the most predominant resource although they did so to differing degrees. The small size and colonial lifestyle of *C. h. hottentotus* allows it to feed almost 100% on geophytes, while the solitary and larger species *G. capensis* and *B. suillus* fed to a greater extent on other resources such as grass and clover. The high reliance by *C. h. hottentotus*, almost 100%, on subterranean food sources such as bulbs and tubers is not only higher than *G. capensis* and *B. suillus* but also higher compared to other subterranean mammals such as the Middle East Blind Mole rat (*Spalax ehrenbergi*), which fed up to 67% on subterranean foods [14] and Attwater’s Pocket Gopher (*Geomys attwateri*) [41] where subterranean foods made up 60% of the diet. *B. suillus,* the largest of the species had the most generalized diet, potentially due to its larger energetic demands coupled with its restriction to sandier looser soils which may further limit the availability of different foods it encounters.

We demonstrate that stable isotope analysis can be applied successfully as a technique to examine dietary patterns in mammals such as mole-rats, where direct observations are impractical, and could be applied in other studies to examine diet choice in subterranean mammals. We also showed the high level of variation that exists between individuals in their diet choices and thus highlight the importance of considering individual variation as well as overall population patterns when assessing diet. Examining dietary patterns and overlap with other sympatric species, at both the inter and intra population level, provides insights into community ecology, increasing our understanding of how species may use resources differently so that they are able to successfully coexist.
